# Preoperative Botulinum Toxin-A Injections Prior to Abdominal Wall Reconstruction Can Lead to Cardiopulmonary Complications

**DOI:** 10.3389/jaws.2024.13433

**Published:** 2024-10-08

**Authors:** W. A. R. Zwaans, A. S. Timmer, M. A. Boermeester

**Affiliations:** ^1^ Department of Surgery, Máxima Medical Center, Veldhoven, Netherlands; ^2^ SolviMáx Center of Excellence for Chronic Abdominal Wall and Groin Pain, Máxima Medical Center, Eindhoven, Netherlands; ^3^ NUTRIM School of Nutrition and Translational Research in Metabolism, Maastricht University, Maastricht, Netherlands; ^4^ Department of Radiology, Amsterdam UMC Location University of Amsterdam, Amsterdam, Netherlands; ^5^ Department of Surgery, Amsterdam UMC Location University of Amsterdam, Amsterdam, Netherlands; ^6^ Amsterdam Gastroenterology Endocrinology and Metabolism, Amsterdam, Netherlands

**Keywords:** botulinum toxins, incisional hernia, abdominal hernia, abdominal wall, abdominal wound closure techniques

Dear Editors,

The majority of incisional hernias are located in the midline of the abdominal wall (linea alba). Repair aims to achieve a complete approximation of the rectus muscles in the midline. Different (surgical) techniques have been described as adjuncts to avoid bridging the hernia defect with mesh, instead of complete closure with native tissue [1–3]. These are so-called component separation techniques (CST). Chemical CST is the most recently described and potentially the most promising development [4, 5]. Chemical CST uses ultrasound-guided deposition of Botulinum toxin-A (BTA) into the lateral abdominal wall muscles to reduce or completely avoid the need for surgical CST [6]. The injections are performed approximately three to 6 weeks before the abdominal wall reconstruction and cause temporary flaccid paralysis of the lateral abdominal wall facilitating closure of the midline of the abdomen during reconstruction [5].

The number of publications on chemical CST is rapidly increasing but (serious) adverse events seem to be largely absent from the literature [7]. Therefore, one may assume that BTA injections do not have any adverse effects (in the short term). We would like to emphasise that although chemical CST has a low risk of complications, it is by no means without risks. Here, we discuss two patients with serious pulmonary complications following preoperative workup with BTA injections prior to abdominal wall reconstruction, and a third patient in whom we refrained from BTA pretreatment. In addition, we suggest some future considerations regarding chemical CST.

An 83-year-old male patient presented to the outpatient clinic with chronic postoperative inguinal pain and suspected chronic infection of a previously implanted lower abdominal mesh. Past medical history included pacemaker implantation, myocardial infarction, laparoscopic right hemicolectomy complicated by multiple intra-abdominal abscesses, open preperitoneal left-sided inguinal hernia repair complicated by a wound infection and subsequent recurrent hernias and incisional hernia repair using mesh in the retrorectus plane. Physical examination revealed a giant inguinal hernia, a small fistula in the left groin, and a 6 cm wide lateral incisional hernia. Additional cross-sectional imaging showed multiple collections of fluid surrounding the giant inguinal hernia, extending from the Anterior Superior Iliac Spine (ASIS) into the scrotum, where the fistula was present. The estimated loss of domain was approximately 25%. The patient was counselled and scheduled for complete mesh removal and abdominal wall reconstruction including reconstruction of the left groin. Prior to surgery, chemical CST was performed, using two bilateral BTA injections (a total of 600 units of Dysport^®^, diluted in 120 mL of 0.9% saline), into all three lateral abdominal wall muscles [8]. Approximately 2 weeks after the injections the patient developed complaints of general fatigue and dyspnoea on exertion. Under the working diagnosis of cardiac decompensation due to mild anaemia (Haemoglobin 109.9 mg/dL; 6.1 mmol/L), and the inability of the lungs to compensate following the botulinum injections, surgery was postponed.

A second patient, a 72-year-old woman, was referred to our tertiary academic centre with a complex incisional hernia. Her past medical history was extensive, and included cystectomy, hysterectomy and sacrocervicopexy with mesh implant, followed some years later by mesh removal for erosion. No chronic obstructive pulmonary disease was diagnosed, although she had 9.25 pack years (having stopped smoking 16 years before). Cross-sectional imaging showed a large lateral incisional hernia with a loss of domain of approximately 20%. Pulmonary testing – performed 14 months earlier – showed an absolute FVC of 2.26 L (relative value 82.11%), FEV1 was measured at 1.63 L (76.08%) and FEV1/VCmax was measured at 72% (i.e., slightly reduced for her age). She underwent preoperative chemical CST with the same dosage, volume and technique as the patient described above. Twelve days after chemical CST she presented to the emergency room with respiratory insufficiency and was diagnosed with and treated for pneumonia. In the following weeks, the infection parameters decreased, but the respiratory weakness remained, and the elective reconstruction was postponed until further notice.

The two cases presented demonstrate that chemical CST prior to abdominal wall reconstruction can result in (short-term) cardiopulmonary complications. A few days after BTA injections, patients may experience adverse effects such as back pain (compensation for truncal stability) or a weakened cough. A case of dyspnoea has been described previously [7]. These effects can be experienced from 2 days after BTA injection, as functional denervation occurs after this period [5]. It is critical to be aware of the potential side effects of BTA injections and to assess preoperative cardiopulmonary function tests to appropriately select patients for this neoadjuvant treatment to avoid these adverse events.

A third patient with a history of multiple sclerosis (MS) and related muscle weakness presented with a large incisional hernia requiring CST for abdominal wall closure. Before surgery was scheduled, she underwent spirometry, which showed moderate pulmonary function. Hence, BTA injections were deemed to be contra-indicated to avoid the previously described complications.

Caution is warranted when using chemical CST in patients with conditions that may influence respiratory muscle function. Asthma and chronic obstructive pulmonary disease (COPD) are common respiratory disorders that restrict respiratory function. In addition to carefully considering the indication for chemical CST, hernia surgeons should always assess pulmonary function (spirometry or cardiopulmonary exercise test; CPET) in pulmonary-impaired patients to determine their eligibility. In patients with neurological/neuromuscular conditions involving neurotransmitter release of acetylcholine, BTA injections are generally considered contraindicated.

The preoperative workup of all patients with complex incisional hernias (with or without enterocutaneous fistulas) includes a complete prehabilitation programme before repair, as recommended by the international hernia societies [9]. Pulmonary training is part of this prehabilitation and is performed by physical therapists in the Netherlands. Before definitive planning of complex incisional hernia repair, patients with a history of cardiopulmonary disease should undergo spirometry and/or CPET. However, to date, no definite cut-off values for spirometry in relation to chemical CST are available. We believe that FEV1 <70% could result in cardiopulmonary problems when BTA injections are performed in the lateral abdominal wall. In our opinion, FEV1 <60% is a relative contra-indication and <50% is an absolute contra-indication for BTA injections prior to complex incisional hernia repair. Caution is warranted as this expert opinion-based recommendation may be subject to new insights when more evidence becomes available in the future.

The transverse abdominal muscle contributes to respiratory function in humans and is preferentially recruited to the superficial muscle layer of the abdominal wall during breathing [10]. Although this muscle is relatively thin compared to the other two lateral abdominal wall muscles, it is known to play an integral role in truncal stability [7]. Based on the two cases presented, the contribution of the transverse abdominal muscle to basic respiration should not be underestimated, particularly in patients with pulmonary disease. A previous study demonstrated some preliminary evidence that applying chemical CST to two (external and internal oblique) muscles instead of the conventional three has similar effects on subsequent fascial closure during repair [7]. It may be questioned whether deviations in chemical CST protocols should be made based on patient comorbidities. Future research should focus on this particular issue before firm conclusions can be drawn.

The physiological changes following chemical CST in lateral and midline complex hernias may be different. The two cases of cardiopulmonary complications of BTA injections described above, involved an incisional hernia outside the midline. As these complications are largely missing from the literature to date, it may be possible that patients with lateral abdominal wall hernias are more likely to develop pulmonary difficulties following chemical CST. As the transversus abdominis muscle has a relatively large contribution to the respiratory function [10], patients with large lateral hernias may have an *a priori* compromised respiratory function compared to the more common patient with a large midline incisional hernia. To date, this statement is solely based on theoretical grounds. Future studies of respiratory function before and after BTA injections could clarify these potential differences and perhaps adjust the preoperative workup.

In conclusion, the use of BTA injections in the lateral abdominal wall is emerging as an excellent surrogate for (or adjuvant to) surgical CST. However, before planning this chemical CST, we advise considering the cardiopulmonary history and neurological disorders that could result in decreased respiratory function, pulmonary functional capacity, and anaerobic threshold, to avoid serious adverse effects of BTA. Pulmonary function tests, like spirometry (in patients with pulmonary comorbidities) and CPET (in patients with cardiopulmonary comorbidities), could give a good indication of whether complications following chemical BTA are plausible. The clinical challenge is to balance the potential postoperative pulmonary advantages of BTA pre-treatment - due to increased abdominal wall laxity and the associated reduced risk of diaphragm elevation - against the potential hazards of reduced axillary respiratory support from the lateral abdominal wall muscles in patients with pulmonary disease.

## Data Availability

The original contributions presented in the study are included in the article/supplementary material, further inquiries can be directed to the corresponding author.
